# Association between life-style behaviors and health outcomes in Adventist and non-Adventist adolescents in Mexico: a pilot study

**DOI:** 10.1186/s12889-019-8042-0

**Published:** 2019-12-19

**Authors:** Maria Elena Acosta Enríquez, Felipe Javier Uribe Salas, Juha Baek, Jenny Patricia Sierra Archbold, Genny Carrillo

**Affiliations:** 1 Montemorelos University, Sciences of Health Faculty, Nutrition School, School of Public Health, Libertad 1300 Pte., C.P. 67500 Montemorelos, Nuevo Leon Mexico; 2 Colegio de la Frontera Norte, Progreso 503, Seccion 1, Amp Tierra y Esperanza, C.P. 26020 Piedras Negras, Coahuila Mexico; 3 Department of Environmental and Occupational Health, School of Public Health, Texas A&M University, 212 Adriance Lab Road, Suite 110, College Station, TX USA

**Keywords:** Adolescents, Life-style behaviors, Health outcomes, Adventist religious affiliation, Mexico

## Abstract

**Background:**

Identifying lifestyle-related health predictors affecting adolescent behaviors is a matter of interest and study for diverse audiences, including the religious sphere. The Adventist religion recommends their followers to adopt a healthy diet, adequate rest, physical activity, sufficient water intake, and non-use of addictive substances such as alcohol, tobacco, and drugs, as well as fostering faith and hope to give meaning to life.

**Methods:**

A cross-sectional and observational study was conducted among adolescent students aged 13 to 19 years old in Montemorelos City, Nuevo León, Northern Mexico, between September 14, 2017 and February 13, 2018. This study included 363 Mexican adolescents, consisting of 202 Adventists and 161 non-Adventists. The binomial logistic regression analysis was performed to examine the relationships between religious affiliation and life-style behaviors and evaluate the effect of life-style behaviors on health outcomes by religious affiliation. Age, gender, type of residence, and place of birth were controlled.

**Results:**

We found that Adventist adolescents were more likely to be watching TV for 2 h or less per day (*p* < 0.001), have enough sleeping time for 7 h or more (p < 0.001), go to bed early at 11 o’clock or before (p < 0.001), and have breakfast (*p* = 0.006) than non-Adventist adolescents significantly. It indicates that Adventist students are more likely to have healthier life-style behaviors than non-Adventist students. The multiple binomial regression models showed that in the group of Adventist adolescents sporting activity and hours watching TV were significantly associated with obesity risk (*p* = 0.001) and risky eating patterns (*p* = 0.044), respectively, controlling for age, gender, type of residence, and place of birth. No relationship was found between life-style behaviors and health outcomes in non-Adventist adolescents.

**Conclusions:**

Religious affiliation could serve as a predictor of healthy behaviors among adolescents. This study concluded that Adventist adolescents are more likely to have a healthier lifestyle behavior than non-Adventist adolescents and various health-related behaviors were specifically identified among Adventist participants.

## Background

The family, social environment, habits, lifestyle, nutrition and health status, and self-perception of body image perception among adolescents demonstrate reasons to identify and understand these behaviors that affect their development, growth, morbidity, and quality of life [1–5]. Various organizations and even religious groups have been interested in adopting health principles that help the reduction of non-communicable diseases. These principles have been observed over time and described in scientific literature, such as those made by the Seventh-day Adventist Church (SDA) since the mid-nineteenth century [6–9]. They have also promoted healthy behaviors such as a vegetarian diet, no alcohol intake, and non-use of tobacco or other addictive substances. In addition, these principles include an appropriate rest, increased recreational physical activity, and development of their faith and hope. Garcia et al. [10] found that Adventist religion principles could influence positively in individual’s physical health, while Miller et al. [11] and Saunders et al. [12] reported a positive effect on mental health.

Previous studies conducted in the Adventist population have examined the impact of lifestyle habits on the prevalence of morbidity and mortality of diseases such as obesity, diabetes, dyslipidemia, heart disease, and cancer [6, 7, 13–16]. During the last 20 years, some researchers have studied the association between religiosity and spirituality in health maintenance and recovery from cross-sectional and longitudinal studies [7, 11, 12, 14–17]. On the other hand, there are few peer-reviewed articles on the relationship between the Adventist religion and the adoption of healthy behaviors in Mexican adolescents. Some unpublished reports evaluated nutritional status, eating habits, body composition, and application of promotional health interventions in children and adolescents [18]. However, other studies have found that healthy lifestyle behaviors are associated with religion and planned activities in Australian [7, 8], Asian [9, 14], Latino [10], American and Czech [19, 20] adolescents.

It has been observed that adolescents and young people involved in some religious affiliation exhibit healthier behaviors, such as participating more actively in extracurricular activities during their free time [21–26] and getting more involved in family and religious social interaction [27, 28] than those who do not. Researchers found that religious affiliation could represent a protective model to reduce obesity and other diseases associated with lifestyle [19], and risks from a history of morbidity [29]. Interventions promoting adolescents’ health should teach them to take care of themselves [30], provide support for parents and their teachers [25, 27], and learn about their social environment and background, in order to be more impactful [13]. The purpose of this study was to compare life-style behaviors between Adventist and non-Adventist adolescents in Mexico and examine the associations between life-style behaviors and health outcomes by religious affiliation.

## Methods

### Participants and settings

A cross-sectional and observational study was conducted among adolescent students aged 13 to 19 years old in Montemorelos City, Nuevo León, Northern Mexico, between September 14, 2017 and February 13, 2018. The study participants were recruited from two middle schools and two high schools, which included one private and one public school, respectively. The population was selected for convenience due to the availability of the subjects to participate in the study. It represents a population cohort that has been evaluated by anthropometry since school age. This study only reports the evaluation data of the last wave of information corresponding to the period of 2017–2018, with a verbal assent from all of adolescent participants and a written consent form from their parents. The study was approved by the Ethics Committee of the Department of Health Teaching and the Health and Research Ministry of in the state of Nuevo León, Registration 194,805,068 and the Regional Office of Education.

### Measurements

The socio-demographic, lifestyle behaviors and health outcomes information were gathered from a non-validated survey questionnaire developed by our research team from direct measurements for height and weight (See Supplementary file). The socio-demographic information included age (years), gender (female or male), place of birth (north Mexico or others), nationality (Mexican or others), school level (middle or high school), type of residence (living with family or living in boarding school), and personal religion (Adventist or non-Adventist). With regards with lifestyle behaviors information, the survey asked participants the following questions: watching TV per a day (hours), sleeping times (hours), time to sleep (o’clock), time to wake up (o’clock), sporting activity (yes or no), and having breakfast (yes or no).

The health outcomes included obesity risk, eating risk scale, and body image self-perception scales. First, the obesity risk was evaluated by using the body mass index (BMI) calculator for child and teen of the Centers for Disease Control and Prevention (CDC) [31]. That is, BMI for each adolescent was calculated as a percentile obtained from a BMI percentile calculator considering age and gender [31, 32]. As a result, the obesity status had four categories as follows: 1) underweight (below the 5th percentile), 2) normal or healthy weight (the 5th to less than the 85th percentiles), 3) overweight (the 85th to less than 95th percentiles), and 4) obesity (the 95th percentile or above) [31]. In the regression models, a binary variable (obese/overweight or normal/underweight) was used to examine a risk of obesity including obese and overweight status.

Second, the eating behavior risk scales of participants were evaluated by using the method of Unikel et al. [33], consisting of a Lickert-style questionnaire with 10 items about the frequency of behaviors during one week (See Supplementary file). Each question took the following values: 0 (never or almost never), 1 (sometimes), 2 (frequently, twice a week), and 3 (very often, more than twice a week). The total score of the scale (0 to 30) was obtained by adding all values of ten questions, indicating the degree of risk in the eating behavior [33]. The following cut-off points were used to evaluate the continuum of the scale: 1) no risk (0 to 6 points), 2) moderate risk (7 to 10 points and 3) high risk (11 or higher points), according to Altamirano et al. [34]. Considering the percentages of each group, we used a binary variable (risk free: 0 to 6 points or having a risk: 7 or higher) in this study.

Lastly, the research team followed the methodology proposed by Gardner et al. and Rueda-Jaimes et al. [35, 36] to evaluate the self-perception about body image among participants. This scale (Standard Figural Stimuli, SFS) consists of 9 silhouettes representing the human figure. The central silhouette indicates the median of weight distribution in the reference population and six silhouettes on left and right sides of the center show progressive increase or decrease in weight. In the questionnaire, participants were asked to select the silhouette that they perceive as their current weight, and the silhouette as their ideal weight. With these measurements two indexes were obtained: a) dissatisfaction index of the body image by calculating the difference between the current perceived image and the desired (or ideal) image, and b) distortion index of the body image by calculating the difference between the perceived image and the real one (real BMI category). In this study, we used satisfaction (satisfaction or dissatisfaction) and distortion (distortion or no distortion) variables as body image self-perception scales.

### Statistical analysis

We calculated descriptive statistics of the study population to estimate mean, standard deviation (SD), minimum (Min) and maximum (Max) values for continuous variables or percentages for categorical variables. The Mann–Whitney U tests for continuous variables such as age, BMI, eating risk scale, hours watching TV, and sleeping time, and the Chi-square tests for categorical variables, including age, gender, place of birth, nationality, school level, type of residence, obesity risk, eating risk scale, dissatisfaction, distortion, hours watching TV, sleeping time per day, time when going to sleep, time to wake up, sporting activity, and having breakfast, were conducted to determine whether socio-demographic characteristics, life-style behaviors, and health outcomes were significantly different between Adventist and non-Adventist adolescent groups.

In addition, the binomial logistic regression analysis was used to figure out the associations between religious affiliation (Adventist or not) and life-style behaviors controlling for age, sex, type of residence, and place of birth. To examine life-style behaviors factors associated with health outcomes by religious affiliation, we used two types of the binomial logistic regression models. The first models controlled for only other life-style behaviors factors and the second models adjusted for other life-style behaviors and some socio-demographic factors, including age, gender, type of residence, and place of birth. The odds ratios (ORs) with 95% confidence intervals (CIs) showed the relationships between individual behavioral factors and each health outcome while controlling for other factors. The “school grade” variable was excluded in the regression models because of its collinearity effect with “age” variable. A *p*-value < 0.05 was considered significant. All analyses were conducted by using Stata version 14 (StataCorp LP, College Station, TX).

## Results

A total of 363 students participated in this study and Adventist and non-Adventist adolescents included 202 (55.6%) and 161 (44.4%), respectively. Descriptive statistics of the study participants about socio-demographic and health-related characteristics are presented in Table 1. The mean age of all participants was 15 years old and Adventist were averagely a little older than non-Adventist students (15.3 vs. 14.7 years, *p* < 0.001). Less than half of participants (44.4%) were under 15 years (13–15 years). Percentages of female and male students were similar but Adventists (51.5%) had more male students than non-Adventists (47.8%). About 62% of students were born in North Mexico and most of participants (92.3%) had Mexican nationality. In addition, over 90% of students were living with family but more Adventists (15.4%) were living in boarding school than non-Adventists (1.9%) (*p* < 0.001).

**Table 1:** Descriptive statistics of study participants in Montemorelos, Nuevo León, Mexico (*N* = 363)

Variable	Total (N = 363)	Adventist (*N* = 202)	Non-Adventist (*N* = 161)	p-value
	Mean ± S.D. [Min, Max] or %			
Age (years)	15.0 ± 1.2 [13,19]	15.3 ± 1.1 [14,19]	14.7 ± 1.3 [13,19]	< 0.001
Age				< 0.001
Less than 15 years old	44.4%	29.2%	63.3%	
15 years old or older	55.6%	70.8%	36.7%	
Gender				0.489
Female	50.1%	48.5%	52.2%	
Male	49.9%	51.5%	47.8%	
Place of birth				< 0.001
North Mexico	62.3%	41.6%	88.2%	
Other^a^	37.7%	58.4%	11.8%	
Nationality				0.034
Mexican	92.3%	91.4%	96.9%	
Other^b^	6.2%	8.6%	3.1%	
School level				< 0.001
Middle school	45.2%	29.2%	65.2%	
High school	54.8%	70.8%	34.8%	
Type of residence				< 0.001
Living with family	90.6%	84.6%	98.1%	
Living in boarding school	9.4%	15.4%	1.9%	
BMI (kg/m^2^)	22.4 ± 4.0 [14.6,36.9]	22.3 ± 3.7 [14.7,35.5]	22.7 ± 4.5 [14.6,36.9]	0.745
Obesity risk				0.076
Underweight/normal	72.2%	75.9%	67.3%	
Overweight/obesity	27.8%	24.1%	32.7%	
Eating risk scale (continuous)	4.3 ± 3.7 [0,22]	4.6 ± 3.8 [0,22]	3.9 ± 3.5 [0.20]	0.06
Eating risk scale				0.144
Risk free (0–6)	80.4%	77.7%	83.8%	
Risk (7 or above)	19.6%	22.3%	16.2%	
Dissatisfaction				0.318
Dissatisfaction	40.8%	56.9%	62.1%	
Satisfaction	59.0%	43.1%	37.9%	0.771
Distortion				
Distortion	71.4%	72.0%	70.6%	
No distortion	28.6%	28.0%	29.4%	

Note: ^a^Central Mexico, South and Southeastern Mexico, and other country; ^b^USA, Central American, South American, and European

In terms of health-related factors, the average BMI of all participants was 22.4 (kg/m^2^) and there was no big difference between Adventists and non-Adventists. The joint prevalence of overweight and obesity was 27.8% and Adventists (24.1%) had lower prevalence than non-Adventists (32.7%). Moreover, the prevalences of risky eating patterns, self-perception body image dissatisfaction and body image distortion were 19.6, 59 and 28.6%, respectively. When comparing Adventists with non-Adventists, Adventist students had more risk eating patterns (22.3% vs. 16.2%), higher satisfaction (43.1% vs. 37.9%), and lower no distortion (28% vs. 29.4%). However, none of the health outcomes showed significant differences between Adventist and non-Adventist adolescents.

Table 2 shows compares life-style behaviors characteristics between Adventist and non-Adventist adolescents. The mean hours watching TV per day was 2.7 h and Adventists (2.2 h) watched TV less than non-Adventists (3.4 h) daily (*p* < 0.001). Percentages of adolescents who watched TV more than 2 h per day were much less in Adventists (22.8%) than non-Adventists (79.5%) (*p* < 0.001). Participants slept for 6.8 h per day averagely and Adventists (7.1 h) had longer sleeping time than non-Adventists (6.4 h) on average (*p* < 0.001). There was a large difference in percentages of students who slept for 7 h or more between Adventists and non-Adventists (80.7% vs. 48.5%) (*p* < 0.001). In addition, percentages of Adventists who went to sleep early (11 PM or before) and woke up late (after 6 PM) were higher than non-Adventists (80.2% vs. 54%, *p* < 0.001; 80.2% vs. 88.2%, *p* = 0.04). The rate of students who attended any sporting activity was higher in non-Adventists than Adventists but the difference was not significant. Lastly, about 71% of adolescents had breakfast and the percentage of those having breakfast was higher in Adventists (78.7%) than non-Adventists (60.9%) (*p* < 0.001).

**Table 2:** Comparisons of life-style behaviors between Adventist and Non-Adventist adolescents

Variable	Total (N = 363)	Adventist (*N* = 202)	Non-Adventist (*N* = 161)	*p*-value
	Mean ± SD [Min, Max] or %			
Hours watching TV (hours)	2.7 ± 1.5 [1,7]	2.2 ± 1.5 [1,7]	3.4 ± 1.2 [1,7]	< 0.001
Hours watching TV per day				< 0.001
Two hours or less	52.1%	77.2%	20.5%	
More than two hours	47.9%	22.8%	79.5%	
Sleeping time (hours)	6.8 ± 1.1 [4,10]	7.1 ± 1.0 [4.3,10]	6.4 ± 1.0 [4,9]	< 0.001
Sleeping time per day				< 0.001
Seven hours or more	66.4%	80.7%	48.5%	
Less than seven hours	33.6%	19.3%	51.5%	
Time when going to sleep				< 0.001
11 PM or before 11 PM	68.6%	80.2%	54.0%	
After 11 PM	31.4%	19.8%	46.0%	
Time to wake up				0.040
6 AM or before 6 AM	83.7%	80.2%	88.2%	
After 6 PM	16.3%	19.8%	11.8%	
Sporting activity^a^				0.698
Yes	60.7%	59.8%	61.8%	
No	39.3%	40.2%	38.2%	
Having breakfast				< 0.001
Yes	70.8%	78.7%	60.9%	
No	29.2%	21.3%	39.1%	

Note: ^a^ Sporting activity includes football, basketball, and volleyball

To examine associations between religious affiliation and life-style behaviors, we conducted multiple binomial logistic regression analysis. The results of the regression models adjusting for age, sex, type of residence, and place of birth are shown in Table 3. We found that Adventist adolescents were more likely to be watching TV for 2 h or less per day (*p* < 0.001), have enough sleeping time for 7 h or more (*p* < 0.001), go to bed early at 11 o’clock or before (*p* < 0.001), and have breakfast (*p* = 0.006) than non-Adventist adolescents significantly. It indicates that Adventist students are more likely to have healthier life-style behaviors than non-Adventist students. The time to wake up and sporting activity did not show significant results.

**Table 3:** Associations between religious affiliation and life-style behaviors

	Adjusted OR (95% CI)					
	Watching TV (2 h or less)	Sleeping time (7 h or more)	Time to sleep (11 o’clock or before)	Time to wake up (6 o’clock or before)	Sporting activity (Yes)	Having breakfast (Yes)
Religion						
Non-Adventist	1 (Ref.)	1 (Ref.)	1 (Ref.)	1 (Ref.)	1 (Ref.)	1 (Ref.)
Adventist	14.3 (7.86–25.87)***	4.37 (2.53–7.57)***	3.8 (2.19–6.70)***	0.83 (0.41–1.68)	1.28 (0.71–2.30)	2.11 (1.23–3.60)**

Note: *OR* Odds Ratio, 95% CI = 95% Confidence Interval, adjusted for age, sex, type of residence, and place of birth

*p<0.05, **p<0.01, ***p<0.001

Table 4 demonstrates the results of associations between life-style behaviors and health outcomes by religious affiliation. The results of binomial logistics regression models showed that sporting activity was significantly associated with obesity risk in the group of Adventist adolescents. That is, students who attended any sporting activity were less likely to have obesity risk (overweight or obesity status) than those who did not attend any sporting activity. The significant results were found in both the model 1 (*p* = 0.019) and model 2 (*p* = 0.001). In addition, we found that hours watching TV had a significant relationship with food risk behavior in the group of Adventist students. Specifically, adolescents who watched TV for 2 h or less per day were less likely to have risky eating pattern than those who watched TV for more than 2 h. Both the model 1 and 2 showed significant results consistently (*p* = 0.044, respectively). However, we did not find any significant results of associations between life-style behaviors and health outcomes in the group of non-Adventist adolescents.

**Table 4:** Associations between life-style behaviors and health outcomes by religious affiliation

Life-style behaviors	Obesity risk		Risky eating patterns		Dissatisfaction		Distortion	
	Adjusted OR (95% CI)		Adjusted OR (95% CI)		Adjusted OR (95% CI)		Adjusted OR (95% CI)	
	Model 1^a^	Model 2^b^	Model 1^a^	Model 2^b^	Model 1^a^	Model 2^b^	Model 1^a^	Model 2^b^
**Adventist adolescents**								
Hours watching TV								
More than 2 h	1 (ref.)	1 (ref.)	1 (ref.)	1 (ref.)	1 (ref.)	1 (ref.)	1 (ref.)	1 (ref.)
2 h or less	2.13 (0.82–5.49)	1.51 (0.55–4.10)	0.45* (0.21–0.98)	0.44* (0.20–0.98)	1.05 (0.52–2.09)	1.11 (0.55–2.25)	1.52 (0.73–3.16)	1.56 (0.73–3.32)
Sleeping time								
Less than 7 h	1 (ref.)	1 (ref.)	1 (ref.)	1 (ref.)	1 (ref.)	1 (ref.)	1 (ref.)	1 (ref.)
7 h or more	0.92 (0.39–2.17)	0.78 (0.31–1.98)	0.73 (0.32–1.67)	0.73 (0.31–1.70)	0.84 (0.41–1.73)	0.82 (0.39–1.73)	1.01 (0.46–2.23)	1.15 (0.51–2.59)
Sporting activity								
No sports	1 (ref.)	1 (ref.)	1 (ref.)	1 (ref.)	1 (ref.)	1 (ref.)	1 (ref.)	1 (ref.)
Sports	0.45* (0.23–0.87)	0.16** (0.06–0.46)	0.98 (0.49–1.99)	1.27 (0.53–.3.01)	0.76 (0.42–1.36)	0.90 (0.44–1.84)	0.62 (0.32–1.20)	0.50 (0.22–1.14)
Having breakfast								
No breakfast	1 (ref.)	1 (ref.)	1 (ref.)	1 (ref.)	1 (ref.)	1 (ref.)	1 (ref.)	1 (ref.)
Breakfast	0.98 (0.43–2.23)	1.01 (0.42–2.41)	0.56 (0.25–1.25)	0.55 (0.24–1.23)	0.98 (0.49–1.98)	0.96 (0.47–1.95)	0.55 (0.23–1.30)	0.56 (0.23–1.32)
**Non-Adventist adolescents**								
Hours watching TV								
More than two hours	1 (ref.)	1 (ref.)	1 (ref.)	1 (ref.)	1 (ref.)	1 (ref.)	1 (ref.)	1 (ref.)
Two hours or less	0.84 (0.34–2.05)	1.20 (0.46–3.15)	1.22 (0.43–3.43)	1.27 (0.43–3.73)	0.58 (0.25–1.31)	0.60 (0.25–1.42)	1.12 (0.45–2.81)	1.07 (0.41–2.80)
Sleeping time								
Less than 7 h	1 (ref.)	1 (ref.)	1 (ref.)	1 (ref.)	1 (ref.)	1 (ref.)	1 (ref.)	1 (ref.)
7 h or more	1.32 (0.66–2.65)	1.35 (0.66–2.77)	0.93 (0.40–2.18)	0.88 (0.37–2.09)	1.87 (0.95–3.69)	1.87 (0.94–3.72)	1.01 (0.49–2.06)	1.05 (0.51–2.18)
Sporting activity								
No sporting activity	1 (ref.)	1 (ref.)	1 (ref.)	1 (ref.)	1 (ref.)	1 (ref.)	1 (ref.)	1 (ref.)
Sporting activity	1.54 (0.73–3.20)	1.53 (0.63–3.68)	1.01 (0.42–2.43)	1.15 (0.42–3.17)	0.87 (0.43–1.73)	0.70 (0.31–1.56)	0.99 (0.47–2.07)	1.02 (0.43–2.42)
Having breakfast								
No breakfast	1 (ref.)	1 (ref.)	1 (ref.)	1 (ref.)	1 (ref.)	1 (ref.)	1 (ref.)	1 (ref.)
Breakfast	0.80 (0.39–1.65)	0.89 (0.42–1.88)	1.00 (0.41–2.42)	1.02 (0.41–2.50)	0.81 (0.40–1.62)	0.77 (0.38–1.57)	1.43 (0.69–2.95)	1.42 (0.68–2.96)

Note: ^a^ Model 1: Adjusted for the other life-style behaviors; ^b^ Model 2: Adjusted for the other life-style behaviors + age, gender, type of residence, place of birth; *OR* Odds Ratio, 95% CI = 95% Confidence Interval

*p < 0.05, **p < 0.01

## Discussion

This study describes the association between Adventist affiliation and lifestyle behaviors among Mexican adolescents living in northeastern Mexico and figure out lifestyle behaviors to affect health outcomes in Adolescent and non-Adolescent students. The results of this pilot study showed that Adventist students were more likely to have healthier life-style behaviors than non-Adventist students. In addition, sporting activity and hours watching TV were found to be significantly associated with obesity risk and risky eating patterns, respectively. Public health literature have identified the most suitable interventions for adolescent populations to adopt healthy behaviors from an early age, and effectively deal with the growing trend of obesity as a primary cause of chronic non-communicable diseases in adolescents [37–39].

Previous studies have showed that religious affiliation could have a protective and motivating effect for adolescents by promoting healthy behaviors to decrease the risk of lifestyle-related diseases [7, 40]. Specifically, a study found that indicators of religiosity and spirituality had positive effects on adolescent health attitudes and behaviors [41]. The other study highlighted the importance of analyzing lifestyle factors on underweight, overweight and obesity prevalence [42]. The findings of these studies support those presented in this study that religious affiliation could affect life-style behaviors among adolescents and several life-style behaviors could be associated with health outcomes.

The result that average hours of watching TV for non-Adventist adolescents was about 3.7 h is consistent with those provided by National Health and Nutrition Survey [43] and the Federal Institute of Telecommunications [44], which reported that children and adolescent Mexicans from 4 to 12 years old watch television for over 4–5 h per day on average. In addition, we found that Adventist students had better sleep habit, including enough sleeping time (7 h or more) and earlier sleep time (11 o’clock or before), than non-Adventist students. This finding is supported by a report from the Sleep Foundation that the teenagers aged 14 to 17 years old are recommended to sleep for 8 to 10 h per day [45].

Our findings showed that Adventist adolescents had lower level of obesity risk (overweight and obesity) than non-Adventist adolescents. This result coincides with those of the previous study that Adventist students demonstrated a lower prevalence of overweight and obesity than non-Adventist population in Australia and the United States [8, 46]. Given that, the prevalence of overweight and obesity is low among the students evaluated when compared with data from the Nuevo Leon region [47]; it is suggested to measure the body composition (lean mass and body fat) to have a more specific criterion of their nutritional status. Furthermore, it is common to motivate Adventists to have breakfast every day considering the lifestyle promotion actions in Adventist institutions. Although this study did not evaluate the type of foods included in breakfast, which can define compliance with the recommendations regarding energy (kcal) and nutrients, the results showed that Adventist students are more likely to have breakfast than non-Adventist students.

However, this study did not find any significant differences in risky eating pattern between Adventist and non-Adventist groups. These results were unexpected in light of the Adventist principle which diet plays an essential theological role [17, 48]. In particular, among the 8515 Adventist schools worldwide [49], these institutions serve vegetarian meals to their students and promote an atmosphere in of balance between diet, exercise and schoolwork [47]. A future study to explore the relationship between diet principle of Adventists and eating risk patterns (diet-related health outcomes) is needed.

Concerning self-reported body image, there were no differences between Adventist and non-Adventist students. Examining body image perception is relevant due to possible positive or negative effects on overall health. Positive self-perception has been related to greater resilience and better academic performance among adolescents [50, 51], whereas a negative body image has been linked to depression and low self-esteem [52, 53].

This study has several limitations. First, the authors were not able to find corresponding themes in the peer-review literature studies performed in Mexico. As a result, they had no previous baseline for comparison among adolescents of Adventist religion in Mexico. In addition, participants live in the same region and locality, which may create a bias. Thus, the results of this study may not be generalized to other population with different characteristics. Future research is needed to include and utilize different regions of Mexico with more participants for comprehensive results. In addition, this study used a self-reported survey, which could make some biases. Lastly, we did not include specific information about life-style behaviors, such as food items for breakfast and times for sporting activity, due to data availability issue. Therefore, it is suggested to specify life-style behaviors to evaluate effects of particular behaviors on health outcomes in future research. However, this study highlights the importance of some lifestyle behaviors and their health impacts between Adventist and non-Adventist adolescents in Mexico. Those behaviors can be predictors of short-term health, as championed by fundamental Adventist principles. It is a pioneering study since it evaluates the uniqueness of Mexican Adventist adolescents, whose religious affiliation provides them the opportunity to be healthy from the earliest stages of life.

## Conclusions

Religious affiliation could serve as a predictor of healthy behaviors among adolescents. This study showed that Adventist students are more likely to have a healthier lifestyle behavior than non-Adventist students. More research is needed to understand lifestyle factors of the Mexican Adventist adolescent population, such as vegetarian diets, daily water consumption, early night rest, outdoor activities, and avoiding alcohol and tobacco consumption. These elements could be identified as predictors of physical health for adolescents in the long term. Given that the prevalence of risky eating behaviors is increasing among adolescents, effective and longitudinal interventions are required to keep adolescents involved and interested in a nutrition and lifestyle program, and consequently reduce short-term risk. The findings of this study suggest expanding this kind of research and it might be needed to individually address each of the adolescents’ life risky behaviors and evaluate their relationships in their family and community.

## Data Availability

The datasets used and analyzed in this study are available from the corresponding author (Dr. Genny Carrillo, gcarrillo@tamu.edu) on reasonable request.

## Abbreviations

BMI: Body Mass Index
CDC: Centers for Disease Control and Prevention
FIT: Federal Institute of Telecommunications
SDA: Seventh day Adventist Church
